# Effects of short physical activity sessions on physical fitness and cognitive control in Norwegian upper secondary school students: the MOVE12 pilot study

**DOI:** 10.1186/s13102-025-01120-7

**Published:** 2025-08-01

**Authors:** Svein Barene, Harald Oseland, Rolf Inge Ølberg, Sigbjørn Litleskare

**Affiliations:** 1 Department of Public Health and Sport Science, University of Inland Norway, Elverum, Norway; 2 Department of Natural Sciences, Practical-Aesthetic, Social and Religious Studies, Østfold University College, Halden, Norway; 3 Department of Public Health, Østfold County Council, Sarpsborg, Norway; 4 Centre for health and technology, University of South-Eastern Norway, Drammen, Norway

**Keywords:** Exercise breaks, High school pupils, Youth, Physical health, Concentration

## Abstract

**Background:**

The MOVE12 pilot study investigated the effects of integrating brief, student-led physical activity sessions on fitness and cognitive control in Norwegian upper secondary school students. The MOVE-break concept integrates strength, endurance, and playful activities into classroom settings to counteract sedentary behavior.

**Methods:**

This 12-week cluster-randomized controlled trial enrolled 517 first-year students from academic and vocational programs. Classes were randomly assigned to either an intervention or control group. The intervention group was encouraged to conduct two daily Move-breaks sessions, each lasting 6–7 min, during classroom instruction. Physical fitness was assessed through aerobic fitness, muscular strength, flexibility, and postural control tests. Cognitive control was evaluated using the Eriksen Flanker and Stroop tasks.

**Results:**

No significant between-group differences were observed in aerobic fitness, muscular strength, flexibility, or postural control over the intervention period. However, within-group improvements were detected in the intervention group for handgrip strength and standing long jump performance. Notably, despite differences in exercise intensity between students in academic and vocational programs, both groups showed significant improvements in cognitive control.

**Conclusions:**

These findings highlight the potential of brief, student-led physical activity sessions to enhance cognitive control in adolescents, irrespective of educational program or intensity levels. While no substantial improvements in physical fitness were observed, the cognitive benefits highlight the potential of integrating structured movement sessions into the school day. Further research should explore optimized intervention strategies and long-term effects on both cognitive and physical health outcomes.

**Trial registration:**

ISRCTN, ISRCTN10405415. Registered 14/12/2023, https://doi.org/10.1186/ISRCTN10405415.

**Supplementary information:**

The online version contains supplementary material available at 10.1186/s13102-025-01120-7.

## Background

In recent decades, there has been a notable decline in physical activity levels among adolescents, coinciding with a rise in sedentary behaviors, particularly due to the increasing use of social media and digital devices [1]. Research indicates that over 80% of adolescents worldwide are not meeting the World Health Organization’s (WHO) recommended levels of physical activity [2], which stipulate at least 60 min of moderate to vigorous activity daily [3]. This trend poses significant concerns, as sedentary lifestyles during adolescence can adversely affect physical, mental, and social health [4]. Physically inactive adolescents are at higher risk for obesity, cardiovascular diseases, and musculoskeletal problems [3, 5]. Socially, reduced physical activity can lead to diminished social interactions and a sense of community, exacerbating feelings of isolation.

The implications of decreased physical activity extend beyond health, affecting academic performance and cognitive control such as attention and concentration [6, 7]. Numerous studies have demonstrated a positive correlation between regular physical activity and enhanced cognitive control, including improved memory, faster information processing, and better concentration [7, 8]. Conversely, high levels of sedentary behavior are linked to poorer academic performance and increased attention deficits [9, 10]. Additionally, the transition to upper secondary school marks a critical period where students face increased academic pressures and social challenges, potentially increasing the risk of school dropout rates [11]. Incorporating physical activity during school hours may potentially mitigate these challenges by improving students’ concentration, reducing stress, and fostering a more engaging and dynamic learning environment.

Schools provide a crucial setting for promoting physical activity, given the substantial amount of time students spend in this environment and the structured nature of the school day. School-based interventions can reach a large and diverse student population, including those from lower socio-economic backgrounds, who tend to have lower physical activity levels than their peers from higher-income families. By integrating physical activity into the school routine, such initiatives can support health promotion without relying on external resources [12]. Moreover, incorporating physical activity into the school day can enhance student engagement, academic performance and school dropout rates.

Despite these benefits, several challenges hinder successful implementation of school-based physical activity programs, such as overcrowded schedules, limited teacher confidence in leading activities, low student awareness of the health risks associated with inactivity, all of which may reduce participation and feasibility [13, 14]. Addressing these barriers is crucial for increasing physical activity levels among school-aged youth [15].

Although evidence from upper secondary classrooms remains limited [20], and systematic reviews report inconsistent effects on physical fitness [21, 22] and cognitive control [23], some studies suggest that aerobic exercise programs may enhance cognitive performance, particularly in attention and executive functions (working memory, cognitive flexibility and inhibitory control) [16, 17]. Furthermore, short bouts of vigorous physical activity, where individuals work at approximately 80% of their maximum heart rate (% of HRmax) for 45 s to 4 min, have been shown to yield similar fitness benefits to longer, lower-intensity workouts [18] and may also positively impact cognitive function and mental health in adolescents [19].

The MOVE12 pilot study evaluates the impact of brief, student-led physical activity breaks on physical fitness and cognitive control in upper secondary students. Specifically, we examine whether students who participate in MOVE-breaks show greater improvements in physical fitness (e.g., aerobic fitness, muscular strength, flexibility and postural control) compared to those who do not receive the intervention. To assess potential acute cognitive effects, we use a within-subject design to determine whether a single 6–7-minute session enhances cognitive control. Given the differences in classroom structure, instructional methods, and student characteristics between academic and vocational programs, we further investigate whether cognitive responses to the intervention differ between these groups. By integrating a scalable, classroom-friendly approach, this study aims to provide practical insights into the potential of structured movement breaks to enhance student well-being and cognitive function in real-world educational settings.

## Methods

### Study design

This 12-week pilot study utilized a cluster-randomized controlled trial (RCT) design to evaluate the effects of a school-based intervention on physical fitness and cognitive control among first-year Norwegian upper secondary school students. Conducted between January and April 2023, the study adhered to the CONSORT 2010 guidelines for reporting randomized trials [20]. Ethical approval was granted by the Research Ethics Committee at Inland University, Norway (Ref. 21/01894), and the trial was registered in the International Standard Randomized Controlled Trial Number Register (ISRCTN10405415) on 14/12/2023. All procedures adhered to the ethical principles outlined in the Declaration of Helsinki.

### Characteristics of the target group

Norwegian upper secondary education has two pathways, academic and vocational. Academic programs emphasize theory for higher education, while vocational programs focus on industry-specific skills through workshops and apprenticeships [21]. Selection depends on academic performance, learning preferences, and social background. Academic students generally have higher grades and stronger educational motivation, whereas vocational students form a more diverse, practice-oriented cohort [21].

Completion rates differ significantly, with 91% of academic students completed upper secondary education between 2017 and 2023, compared to 71% of vocational students, indicating higher dropout rates in vocational programs [22]. Moreover, academic students lead healthier lifestyles, engaging in more physical activity, while vocational students exhibit greater sedentary behavior, poorer diets, and higher tobacco and alcohol consumption [23, 24, 25, 26]. These disparities may increase long-term health risks, highlighting the need for targeted public health interventions.

### Recruitment of participants

**Fig. 1:**
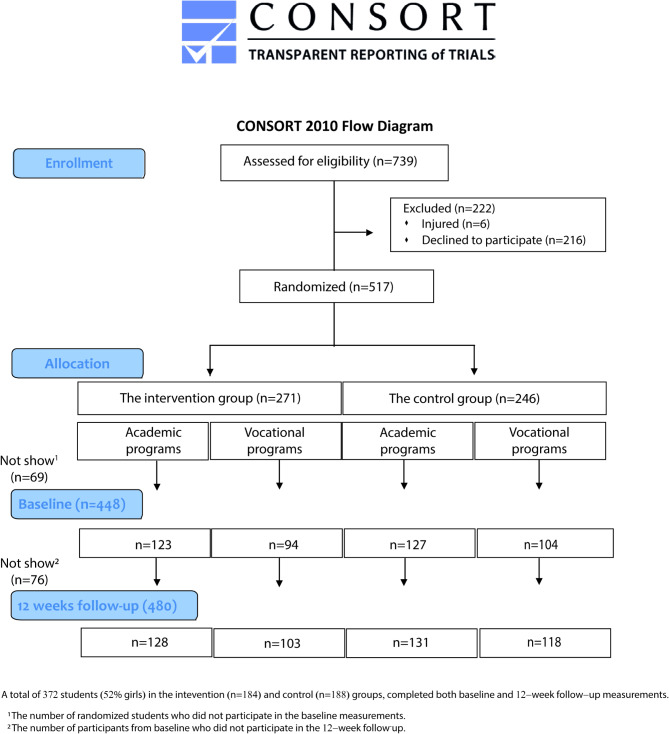
Flow chart illustrates the number of students initially enrolled in the study, their randomization into intervention and control groups, and the number of dropouts at 12-week follow-up

The target population comprised first-year upper secondary students of any gender, aged 16 to 17, from a broad geographic region in Eastern Norway encompassing three counties. Our aim was to recruit a balanced mix of schools offering both academic and vocational programs. Invitations were extended to all 27 upper secondary schools within the area, and five schools agreed to participate, three offering academic programs and two offering vocational programs. One of the vocational schools, significantly larger than the others, contributed approximately 200 students, while the remaining four schools contributed around 100 students each. In total, 739 students were eligible, with 519 consenting to participate (see CONSORT flow chart, Fig. 1).

### Randomization procedure

At each of the five participating schools, classes were randomly assigned to either the intervention group or the control group using a 1:1 allocation ratio. This process was conducted through a blinded lot-drawing method by the principal investigator and project leader, ensuring impartial assignment. At the three schools offering academic programs (class sizes: *n* = 25–30), school administrators first selected four classes with the most homogeneous characteristics. The randomization involved two boxes: one containing group labels (intervention or control) and the other containing class numbers (1, 2, 3, or 4). The process began with drawing a group label from the first box, followed by drawing a class number from the second box. The next draw from the second box was then automatically assigned to the remaining group in the first box. This process was repeated until all classes were allocated to either the intervention or control group. For the two schools offering vocational programs, where class sizes are smaller (*n* = 12–17), a stratified approach was used in collaboration with school management to ensure that classes were as homogeneous as possible, matched by gender, class size, and subject area. The stratified classes were placed into four separate boxes (A, B, C, or D). Randomization followed the same procedure as for the general studies specialization, ensuring equal distribution between the intervention and control groups.

### Intervention content

The 12-week intervention integrated structured physical activity into regular classroom sessions through MOVE-breaks, 6–7-minute activity session encouraged to be conducted twice daily. Students, working in pairs, designed and led these sessions as part of a mandatory physical education assignment, which required them to plan, implement, and evaluate peer-led physical activities.

To support this, the project team developed a digital activity guide featuring strength- and endurance-based exercises tailored for classroom settings, with images and descriptions for easy implementation. Students also had access to classroom projectors and sound systems for streaming follow-along dance videos (e.g., Just Dance on YouTube). Simple equipment, such as beanbags, playing cards, and a large die, was provided to facilitate basic games and team activities.

Before the intervention, students were informed that MOVE-breaks aligned with a physical education competency goal. They participated in two 90-minute practical sessions, where they worked in pairs to design, implement, and assess MOVE-breaks using the activity guide under teacher supervision. These sessions took place in classrooms or nearby indoor/outdoor spaces. Additionally, a 5-minute promotional video was created to inform teachers about the benefits of integrating MOVE-breaks into classroom routines.

Students in the control group were encouraged to maintain their usual activity levels and lifestyle. Beyond their regular physical education classes, they were not offered any additional physical activity sessions or structured exercise breaks. This approach aimed to minimize external influences on activity levels while allowing for a more focused evaluation of the intervention’s effects.

### Pre and post intervention assessments

At baseline (January 2023) and at the 12-week follow-up (April 2023), all participants from both the intervention and control groups completed a series of physical fitness tests. These assessments were conducted in the school’s sports hall by a research team of three researchers, assisted by two of the school’s physical education teachers. which were conducted in the sport hall of their respective schools.

#### Aerobic fitness

Aerobic fitness was evaluated using the YMCA 3-minute step test. Participants were equipped with a Polar Verity Sense (Polar Electro Oy, Kempele, Finland) optical heart rate monitor, placed on their right upper arm. The test was performed barefoot and involved stepping up and down a 30 cm step box at a pace of 96 beats per minute, guided by a metronome, for a total duration of 3 min. Immediately following the stepping exercise, participants remained seated on the step box for a 1-minute recovery period. The recovery heart rate was calculated as the difference between the heart rate measured immediately after the exercise and the heart rate recorded at the end of the 1-minute recovery period [27]. This value was documented to assess participants’ aerobic fitness levels. Changes in recovery rate (bpm) were compared pre- and post-intervention, with a greater reduction in bpm indicating an improvement in aerobic fitness.

#### Upper and lower limb strength

The standing long jump test was performed barefoot on an indoor long jump mat made of non-slip PVC (Sport-Thieme, Lower Saxony, Germany), marked in 5 cm increments ranging from 1 to 3 m. Participants were instructed to execute a standing long jump, beginning from a semi-squat position while utilizing arm swings to generate momentum. The starting point was indicated by a line on the mat. Two testers were involved in the measurement process: the primary tester, who stood near the landing area to ensure precise measurements, and an assistant who recorded the results at a nearby table. Each participant was given three attempts, with the longest jump measured to the nearest centimeter and recorded as the final score. The measurement was taken from the starting line to the point where the rearmost heel landed. If a participant fell backward, the distance was measured from the starting line to the closest body part that touched the ground. A rest period of approximately two minutes was provided between each jump.

Handgrip strength was assessed using a hydraulic hand dynamometer (Baseline, White Plains, NY, USA). Participants were seated at the edge of a chair, holding the dynamometer in their dominant hand. The upper arm was kept vertical, and the elbow was bent at a 90-degree angle. Participants were instructed to squeeze the dynamometer with maximum isometric effort for 5 s, while avoiding any additional body movement. Three attempts were allowed for each participant, with approximately two minutes of rest between each attempt. The test administrator encouraged participants to exert maximum effort during each trial. The highest reading from the three attempts was recorded as the final handgrip strength.

#### Flexibility

Flexibility in this study was evaluated using the ‘sit and reach’ test, a widely accepted method effective for participants of all ages [28, 29, 30]. During the test, participants sat barefoot on the floor with their legs extended straight and placed the soles of their feet against a box, positioned at the zero mark. Measurements were taken in centimeters, recorded as either negative or positive relative to the zero point, depending on the reach. Participants reached forward smoothly, maintaining the stretch for 1–2 s to ensure accuracy, with their knees kept straight and hands overlapped and flat against the measure.

#### Postural control

Postural control was assessed using a force platform (FP4, Hur Labs Oy, Tampere, Finland) equipped with its associated software. Participants underwent two balance tests: a bipedal eyes-closed (EC) test and a unipedal eyes-open (EO) test. Instructions were to maintain stillness with arms crossed and hands on opposite shoulders for 30 s. In the unipedal EO test, participants positioned the free leg’s big toe against the medial malleolus of the standing leg and fixed their gaze on a mark 2 m away [31]. Each test allowed up to two retries if balance was not maintained for the full duration. The tests were timed, conducted without shoes, and a 30-second rest was given between tests. An experienced assessor administered both the initial and retest sessions. Trace length was recorded for the full 30 s and used for further analysis as recommended [32].

### Mid-intervention assessments

At the midpoint of the intervention (March 2023), additional assessments were conducted exclusively on students in the intervention groups. To evaluate the acute effects of a single 6–7-minute MOVE-break session on cognitive control, we employed a within-subject design, where participants served as their own controls. Pre- and post-measurements were conducted with a minimum three-hour interval, following Millisecond Software recommendations [33] to mitigate potential learning effects. During these assessments, intensity measurements were also collected to examine whether cognitive responses were linked to exercise intensity or occurred independently of activity level.

#### Cognitive control

Cognitive control refers to complex cognitive processes essential for focused attention, goal-directed behavior, and inhibiting automatic impulses [34]. It was assessed using two computerized tasks, administered via Inquisit 6 software on students’ smartphones [33] immediately before and after a 7-minute MOVE-break session. The Eriksen Flanker Task, lasting approximately 3 min, evaluated the ability to suppress irrelevant dominant responses. Participants viewed five arrows, with the central arrow inside a box. They were tasked with identifying whether the central arrow pointed left or right. The surrounding arrows (flankers) either pointed in the same direction (congruent trials) or the opposite direction (incongruent trials) [35]. The Stroop Test, lasting about 2 min, required participants to identify the color of a word on the screen, ignoring its written meaning. The test comprised congruent trials (word and font color matched), incongruent trials (word and font color differed), and control trials with colored rectangles [36].

#### Heart rate measurements, rating of perceived exertion and enjoyment

During the 7-minute MOVE-break session, the intensity levels were tracked for students in the intervention classes. Heart rate was recorded at 5-second intervals using Polar heart rate monitors (POLAR Team System, Polar Electro, Kempele, Finland). The data were transferred to a PC via a docking station and processed using Microsoft Excel (Microsoft Corporation, 2018).

To assess students’ self-perceived exertion, they completed a questionnaire immediately after the session, rating their exertion on a 1–10 visual analogue scale (VAS) (1 = ‘No exertion at all’, 10 = ‘Maximal exertion’): (1) “How physically demanding did you find today’s MOVE-break?” [37], (2) “How much strain did today’s MOVE-break place on your legs?” [38, 39], and (3) “How breathless were you during today’s MOVE-break?” [40]. The internal consistency of these three measures was evaluated using Cronbach’s alpha, yielding a reliability coefficient (α) of 0.86, indicating high internal consistency and supporting their reliability as indicators of perceived exertion during the MOVE-break session.

Additionally, to assess students’ subjective enjoyment and its relationship with physiological responses and perceived exertion, students rated their enjoyment on a 1–10 visual analogue scale (VAS) by answering: “How enjoyable did you find today’s MOVE-break?”

### Intervention adherence

The adherence rate was retrospectively assessed through an electronic questionnaire at the 12-week follow-up using the following question: “On average, how often did your class perform MOVE-breaks during the 12-week study period?“. Responses were recorded on a scale ranging from 1 (engagement only a few times during the study period), 2 (less than once per week), 3 (2–4 times per week), 4 (approximately once per day), 5 (approximately twice per day), and 6 (more than twice per day).

### Statistical analysis

A priori sample size calculations using G-Power [41], indicated that a total of 352 participants is required to detect a moderate effect size (d = 0.3). This calculation was based on a two-group design, with 80% power and a significance level (alpha) of 0.05.

All statistical analyses in this study were conducted using STATA version 21.0. A linear mixed model was employed to evaluate both between-group differences and within-group changes from baseline to the 12-week follow-up, incorporating study track program as fixed effects and subject-specific random effects to enhance model accuracy. This approach effectively handles repeated measures, accounts for within-subject correlations, and accommodates missing data, ensuring a robust analysis of the intervention’s impact on physical fitness and cognitive control over time [42]. We applied the restricted maximum likelihood estimation method, with degrees of freedom adjusted via the Satterthwaite approximation.

Acknowledging that gender differences in physical fitness tend to become more pronounced during adolescence [43], with boys typically experiencing greater increases in VO_2_max and muscle mass [44], we conducted subgroup analyses to examine potential gender-specific variations in intervention-related changes in physical fitness outcomes. Additionally, subgroup analyses were performed for all physical fitness outcome measures across the two respective study program tracks, based on previously reported differences in student characteristics between these groups (see Subsection 2.2).

Effects on outcome variable effects were determined from least square means (overall means) with 95% confidence intervals and *p*-values. Cognitive control outcomes were analyzed using paired t-tests, while baseline characteristics, intensity levels, rating of perceived exertion, and enjoyment during Move-break sessions across study tracks, were compared using two sample t-tests. Statistical significance was set at *p* < 0.05, with effect sizes for significant findings calculated using Cohen’s d.

## Results

### Baseline characteristics

A total of 517 students provided written consent to participate in the study. Of these, 448 (boys: *n* = 208, girls: *n* = 233, unspecified: *n* = 7) completed baseline measurements in January 2023. At the 12-week follow-up in April 2023, 480 students (boys: *n* = 218, girls: *n* = 255, unspecified: *n* = 7) participated. To enable gender-specific subgroup analyses, individuals in the unspecified category were excluded from the statistical sample.

At baseline, participants had an average age of 16.2 ± 0.5 years, height of 171.6 ± 9.7 cm, weight of 67.1 ± 14.9 kg, and BMI of 22.7 ± 4.2 kg/m² (Table 1). No significant between-group differences were observed for any of the baseline outcome variables.

**Table 1:** Baseline characteristics of age, anthropometry, aerobic fitness (heart rate recovery), upper and lower limb strength, flexibility, and postural control for the intervention group and the control group

Characteristics		Intervention group (*n* = 213)		Control group (*n* = 228)		Total (*n* = 441)		*P*-values
		Mean	SD	Mean	SD	Mean	SD	
Age (years)		16.3	0.6	16.2	0.5	16.2	0.5	0.137
Height (cm)		171.3	9.8	172.0	9.7	171.6	9.7	0.462
Weight (kg)		67.7	15.5	66.5	14.3	67.1	14.9	0.398
BMI (kg/m^2^)		23.0	4.3	22.4	4.2	22.7	4.2	0.177
Aerobic fitness (HRR)		39.7	12.1	39.5	11.4	39.6	11.7	0.874
Upper and lower limb strength								
	Hand grip strength (kg)	41.9	12.6	40.8	12.3	41.3	12.5	0.343
	Standing long jump (cm)	175.3	34.2	178.1	35.4	176.7	34.8	0.395
Sit and reach flexibility (cm)		3.3	10.2	3.1	10.2	3.2	10.2	0.813
Postural control (trace length)								
	Bipedal, eyes closed (mm)	715.6	226.8	726.7	223.2	721.4	224.8	0.607
	Unipedal, eyes open (mm)	1196.3	422.8	1205.8	413.2	1201.2	417.4	0.812

HRR = heart rate recovery

### Intervention adherence

Among the 153 participants in the intervention group, retrospective questionnaire data were available for 125 (82%). The median adherence score was 4, corresponding to an average implementation rate of one session per day.

### Physical fitness

**Fig. 2:**
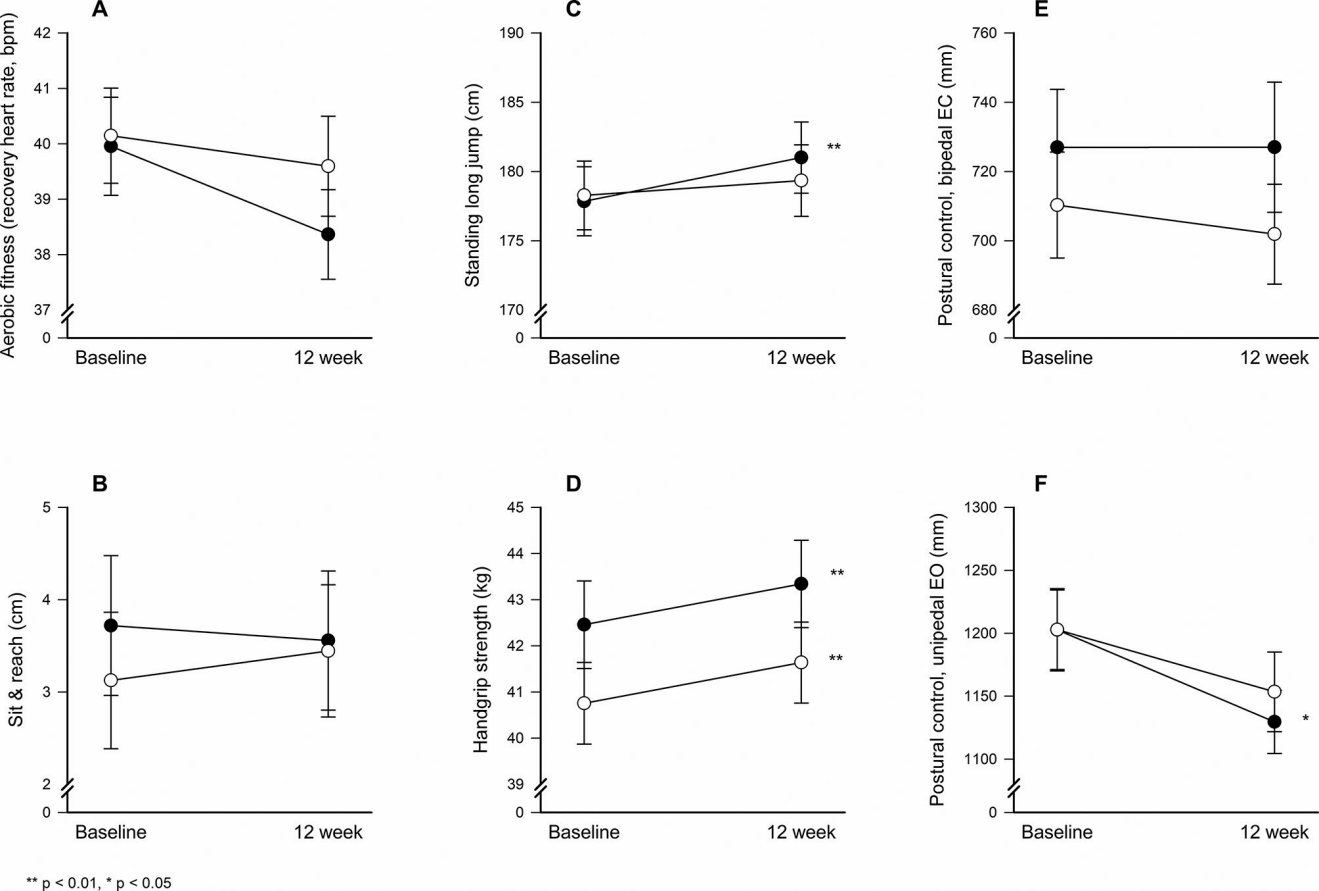
Within-group changes in aerobic fitness (**A**), flexibility (**B**), lower limb strength (**C**), upper limb strength (**D**), postural control, bipedal with eyes closed (**E**), and postural control, unipedal with eyes open (**F**) in the intervention group (●) and the control group (○). Error bars are presented as standard errors (SE)

Linear mixed model analyses revealed no significant between-group differences in physical fitness measures from baseline to the 12-week follow-up (Table 2). However, within the intervention group, small but statistically significant improvements were observed in standing long jump (3.3 cm, 95% CI 1.4 to 5.1, *p* = 0.001, d = 0.09), handgrip strength (0.9 kg, 95% CI 0.2 to 1.5, *p* = 0.007, d = 0.07) and unipedal stance with eyes open (-65.9 mm, 95% CI -122.3 to -9.4, *p* = 0.022, d = 0.19) (Fig. 2). Similarly, the control group exhibited a small yet significant within-group improvement in handgrip strength (0.9 kg, 95% CI 0.2 to 1.5, *p* = 0.007, d = 0.07).

**Table 2:** Within-group changes and between-group differences in body mass index (BMI), aerobic fitness (measured by recovery heart rate), upper and lower limb strength, postural control and flexibility (assessed via the sit-and-reach test) from baseline to 12-week follow-up among study participants (*n* = 473; 54% girls), including the intervention group (*n* = 227; 53% girls) and the control group (*n* = 246; 54% girls)

		Within-group changes						Between-group differences		
Outcome measure		Intervention groups			Control group					
		Diff	95% CI	*p*-value	Diff	95% CI	*p*-value	Diff	95% CI	*p*-value
BMI (kg/m^2^)		0.1	-0.0–0.3	0.060	0.1	-0.0–0.3	0.129	0.6	-0.1–1.4	0.108
Aerobic fitness (bpm)		-1.3	-3.1–0.5	0.144	-0.4	-2.1–1.4	0.681	-0.1	-1.9–1.7	0.884
Upper and lower limb strength										
	Handgrip strength (kg)	0.9	0.2–1.5	0.007	0.9	0.2–1.5	0.007	0.8	-1.4–3.0	0.465
	Standing long jump (cm)	3.3	1.4–5.1	0.001	1.1	-0.8–2.9	0.249	-2.3	-8.6–4.0	0.469
Sit and reach test (cm)		-0.0	-0.8–0.7	0.887	0.4	-0.3–1.1	0.306	-0.2	-2.0–1.6	0.852
Postural control (trace length)										
	Bipedal, eyes closed (mm)	4.0	-22.3–29.2	0.759	-18.0	-42.8–6.9	0.156	6.4	-31.6–44.3	0.743
	Unipedal, eyes open (mm)	-65.9	-122.3 – -9.4	0.022	-50.1	-105.6–5.4	0.077	-13.5	-79.9–52.8	0.689

Within-group data are presented as mean change (95% CI) and between-group data as estimated overall mean difference (95% CI)

Consistent with the main analysis, gender-specific subgroup analyses revealed no significant improvements in aerobic fitness for either boys or girls. However, subgroup analysis of the standing long jump indicated that the within-group improvement in the intervention group was primarily driven by boys (5.3 cm, 95% CI 2.4 to 8.1, *p* < 0.001, d = 0.18), with no significant change among girls (*p* = 0.180). In contrast, subgroup analysis of handgrip strength revealed that the intervention group’s improvement was mainly attributable to girls (1.5 kg, 95% CI 0.8, 2.2, *p* < 0.001, d = 0.27), with no significant increase among boys (*p* = 0.610). In the control group, both girls (0.9 kg, 95% CI 0.2, 1.6, *p* = 0.013, d = 0.26) and boys (1.1 kg, 95% CI 0.0, 2.2, *p* = 0.046, d = 0.10) demonstrated significant within-group improvements in handgrip strength.

### Impact of intensity levels on cognitive control

**Fig. 3:**
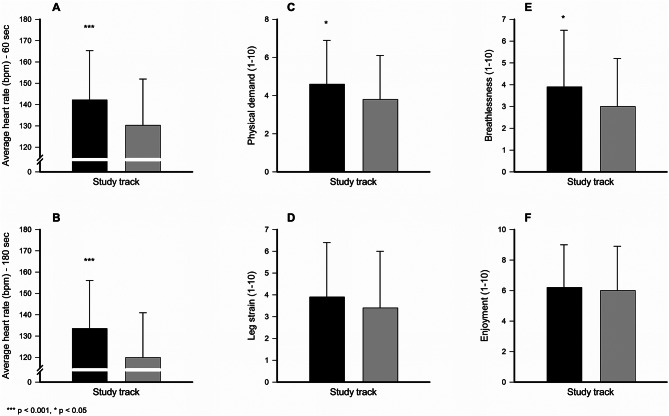
Average heart rate levels over 60-second (**A**) and 180-second intervals (**B**), self-perceived physical exertion (**C**), leg strain (**D**), breathlessness (**E**), and enjoyment (**F**) during participation in a specific MOVE-break session, categorized by academic track (black bars) and vocational track (gray bars). Error bars are presented as standard deviations (SD)

As shown in Table 3, students in academic programs exhibited significantly higher heart rates than vocational students at both the 60-second (142.2 ± 23.1 bpm vs. 130.3 ± 21.7 bpm, *p* < 0.001, d = 0.54) and 180-second (133.5 ± 22.6 bpm vs. 119.9 ± 21.0 bpm, *p* < 0.001, d = 0.63) intervals, indicating a greater physiological response during the MOVE-break session (Fig. 3).

**Table 3:** Students’ average heart rate (bpm) and ratings on a 1–10 visual analogue scale of self-perceived physical demand, leg strain and breathlessness, as well as their perceived enjoyment during a MOVE-break session in the intervention group (*n* = 156), stratified by academic (*n* = 91) and vocational (*n* = 65) programs

Characteristics		Academic programs		Vocational programs		Diff.	*p*-value
		Mean	SD	Mean	SD		
Average heart rate (bpm)							
	60 s Interval	142.2	23.1	130.3	21.7	11.9	0.000
	180 s Interval	133.5	22.6	119.9	21.0	13.6	0.000
Perceived physical demand (1–10)		4.6	2.3	3.8	2.3	0.8	0.025
Perceived leg strain (1–10)		3.9	2.5	3.4	2.6	0.5	0.160
Perceived breathlessness (1–10)		3.9	2.6	3.0	2.2	0.8	0.024
Perceived enjoyment (1–10)		6.2	2.8	6.0	2.9	0.2	0.764

Since maximum heart rate (HRmax) was not directly measured, intensity levels were estimated using the Tanaka equation (HRmax = 208–0.7 × age) [45], yielding an approximate HRmax of 196 bpm for 16–17-year-olds. Based on this estimation, the highest recorded 180-second interval heart rates, 133 bpm for academic students and 120 bpm for vocational students, corresponded to approximately 68% and 61% of HRmax, respectively. According to established exercise intensity classifications [46], these values indicate that academic students exercised at a moderate-to-vigorous intensity level, while vocational students remained within the moderate intensity range.

Regarding perceived exertion, students in academic programs reported significantly higher physical demand (4.6 ± 2.3 vs. 3.8 ± 2.3, *p* = 0.025, d = 0.35) and breathlessness (3.9 ± 2.6 vs. 3.0 ± 2.2, *p* = 0.024, d = 0.34) than their vocational counterparts (Table 3). However, no significant differences were observed in leg strain (*p* = 0.160) or perceived enjoyment (*p* = 0.764) between groups. These findings suggest that students in academic programs experienced greater physiological and self-perceived exertion during the MOVE-break session, while enjoyment levels remained comparable.

**Fig. 4:**
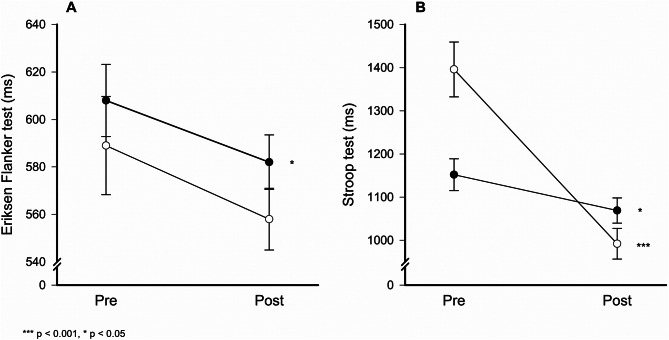
Changes in cognitive control measured pre (without prior physical activity) and post (immediately after participation in a specific MOVE-break session) using the Eriksen Flanker test (**A**) and Stroop test (**B**), categorized by academic track (●) and vocational track (○). Error bars are presented as standard errors (SE)

Despite variations in heart rate during MOVE-break session, both academic and vocational students demonstrated notable improvements in cognitive control (Table 4). In the Eriksen Flanker test, academic students showed a small but significant improvement (t(87) = 2.13, *p* = 0.036, d = 0.21), while vocational students showed only a trend toward improvement (t(60) = 1.92, *p* = 0.059, d = 0.22). In the Stroop test, academic students achieved a moderate effect (t(90) = 2.55, *p* = 0.012, d = 0.26), whereas vocational students demonstrated a large improvement (t(64) = 8.32, *p* < 0.001, d = 0.97) (Fig. 4).

These findings underscore that even brief physical activity sessions can significantly enhance attention-related cognitive control, with varying degrees of impact across educational tracks.

**Table 4:** Within-group changes in cognitive control assessed through Eriksen flanker and Stroop test performance, along with intensity levels measured by average heart and corresponding rating of perceived exertion (RPE) on a 1–10 visual analogue scale during a MOVE-break session, in the intervention group (*n* = 156), stratified by academic (*n* = 91) and vocational (*n* = 65) programs

Characteristics	Academic programs (*n* = 91)						Vocational programs (*n* = 65)					
	Pre		Post		Diff.	*p*-value	Pre		Post		Diff.	*p*-value
	Mean	SD	Mean	SD			Mean	SD	Mean	SD		
Cognitive control												
Eriksen Flanker test (ms)	608	145	582	110	26	0.036	589	167	558	105	31	0.059
Stroop test (ms)	1152	351	1069	279	83	0.012	1396	512	992	286	406	0.000

^†^ ms = milliseconds, bpm = beats per minute

## Discussion

The MOVE12 pilot study investigated the effects of brief, student-led physical activity sessions integrated into the school day on various physical fitness and cognitive control among upper secondary school students. Given the global decline in adolescent physical activity levels and the associated health risks [2, 4], understanding effective intervention strategies within educational settings is crucial. This discussion evaluates the study’s findings concerning aerobic fitness, muscular strength, flexibility, postural control, and cognitive control, contextualizing them within existing literature and highlighting implications for future interventions.

### Physical fitness

The intervention did not yield significant between-group differences in aerobic fitness, as measured by the YMCA 3-minute step test. This result is consistent with previous research suggesting that short-duration, moderate-intensity interventions may be insufficient to induce substantial improvements in cardiovascular endurance among adolescents [47, 48, 49]. Studies indicate that higher-intensity and longer-duration activities are more effective in enhancing aerobic capacity in this population [50, 51]. Our intensity analyses revealed that students in the academic programs reached moderate-to-vigorous intensity levels, while those in the vocational programs remained within moderate intensity range. Given this, the MOVE-breaks likely did not provide the necessary stimulus for significant cardiovascular adaptation.

The findings on muscular strength measured by handgrip strength and standing long jump performance, were mixed. No significant between-group differences were observed for either measure, suggesting that the MOVE-break concept may lack sufficient resistance-based exercises or that student engagement in these exercises was insufficient to induce meaningful improvements in muscular performance, particularly in maximal force and explosive power. These findings align with a systematic review by García-Baños et al. (2020), which concluded that significant strength gains occurred only when structured resistance exercises were integrated into physical education programs [52]. Similarly, a review by Cox et al. (2020) reported low-to-moderate effects of school-based interventions aimed at improving muscular fitness in boys [53]. However, evidence suggests that interventions incorporating targeted strength training and plyometric exercises yield significant improvements in muscular strength and power, highlighting the importance of exercise specificity and intensity in achieving measurable performance gains [54].

Regarding standing long jump performance, a significant within-group improvement (3.3 cm, 95% CI 1.4 to 5.1, *p* = 0.001, d = 0.09) was observed in the intervention group, indicating potential enhancements in lower-body explosive strength attributable to the MOVE-breaks. Nonetheless, the lack of between-group differences warrants caution in attributing these gains solely to the intervention. Gender-specific subgroup analyses revealed that this improvement was exclusively to boys (5.3 cm, 95% CI 2.4 to 8.1, *p* < 0.001, d = 0.18). This finding aligns with previous research showing that boys consistently outperform girls in standing long jump throughout childhood and adolescence, with performance differences becoming more pronounced after the age of 14 due to pubertal hormonal changes that contribute to increased muscle mass and strength [55, 56].

In terms of handgrip strength, identical small significant within-group improvements (0.9 kg, 95% CI 0.2 to 1.5, *p* < 0.007, d = 0.07) were revealed in both groups, suggesting that factors beyond the intervention, such as natural growth, maturation, or extracurricular activities, may have contributed to these gains [57]. Gender-specific subgroup analyses revealed moderate significant improvements in handgrip strength for girls in both the intervention group (1.5 kg, 95% CI 0.8, 2.2, *p* < 0.001, d = 0.27) and the control group (0.9 kg, 95% CI 0.2, 1.6, *p* = 0.013, d = 0.26). In terms of boys, the control group showed small but significant improvements in hand grip strength (1.1 kg, 95% CI 0.0, 2.2, *p* = 0.046, d = 0.10), with no such improvement in the intervention group. This finding is particularly noteworthy, given that previous research has generally reported higher maximal isometric handgrip strength in boys compared to girls [58, 59, 60]. The observed gender-specific improvement favoring girls is likely attributable to differences in baseline strength levels [61], rather than variations in engagement with specific movement patterns.

Flexibility, assessed via the sit-and-reach test, did not show significant changes in either group. This finding is consistent with existing literature suggesting that flexibility improvements require targeted stretching exercises performed regularly over extended periods [62, 63]. The lack of improvement suggests that the MOVE-breaks did not include sufficient flexibility-focused activities or were too brief to elicit meaningful adaptations.

Postural control measures did not show significant improvements following the intervention. Maintaining and improving balance requires targeted exercises that challenge the vestibular and proprioceptive systems [64, 65]. The general physical activities included in the MOVE-breaks may not have sufficiently stressed these systems to produce observable changes.

Future research should consider incorporating longer or more intense activity sessions to effectively target aerobic fitness, integrating dedicated stretching components into school-based activity sessions to promote flexibility development, and including balance-specific exercises, such as single-leg stands or dynamic stability tasks, to enhance postural control outcomes. Additionally, standardizing exercise protocols and ensuring consistent implementation across educational settings would help clarify intervention effects.

### Cognitive control

A notable finding of this study was the significant improvement in cognitive control observed among both academic and vocational program students following the MOVE-break sessions, as demonstrated by enhanced performance on the Eriksen Flanker and Stroop tests. While academic program students exhibited a small but significant improvement in the Eriksen Flanker test (t(87) = 2.13, *p* = 0.036, d = 0.21) and a moderate effect in the Stroop test (t(90) = 2.55, *p* = 0.012, d = 0.26), vocational program students only showed a tendency for improvement in the Eriksen Flanker test (t(60) = 1.92, *p* = 0.059, d = 0.22) and a large effect in the Stroop test (t(64) = 8.32, *p* < 0.001, d = 0.97). These results align with existing evidence suggesting that acute bouts of physical activity can enhance cognitive control, including attention and executive processing [7, 66]. The observed cognitive benefits may be attributed to increased cerebral blood flow, neurochemical changes, and enhanced synaptic plasticity induced by physical exertion [67]. Notably, these improvements occurred regardless of the slight differences in activity intensity between the two educational tracks, suggesting that even moderate-intensity exercise can benefit cognitive control. These findings underscore the potential of integrating short physical activity breaks into academic schedules as a practical and effective strategy to support students’ cognitive functioning and learning outcomes across diverse educational contexts.

### Strengths and limitations

This study’s randomized controlled design and large sample size enhance the credibility and generalizability of the findings, while the real-world school setting adds practical relevance. However, several limitations should be noted. The lack of standardized exercise protocols likely contributed to inconsistent outcomes, and the absence of compliance data limits the ability to link participation levels with results. Implementing monitoring systems like attendance tracking could clarify these relationships in future studies. Additionally, confounding variables such as extracurricular activities and varying baseline fitness levels may have influenced the results. The short intervention period also limits the assessment of long-term effects, suggesting the need for extended interventions and follow-up assessments. Lastly, self-selection bias might have impacted participant engagement, highlighting the importance of addressing this in future research. Addressing these limitations will help refine intervention strategies and improve their effectiveness in promoting adolescent health.

## Conclusions

While the intervention had a limited impact on broader physical fitness measures, it demonstrates that even brief bouts structured physical activity can enhance cognitive control, thereby supporting students’ mental and academic performance. These findings highlight the importance of well-designed, systematically implemented school-based physical activity interventions to address adolescent physical inactivity while promoting cognitive health and academic success. Future research should focus on refining intervention strategies by incorporating more structured and systematically guided activity content to better understand and interpret potential training adaptations and physiological effects. Additionally, enhancing adherence monitoring systems is essential for improving implementation fidelity and accurately assessing student engagement. Examining the long-term impacts of such interventions will provide valuable insights into their sustained benefits for both physical and cognitive health. Standardizing exercise protocols and ensuring consistent implementation in upper secondary school settings could further clarify the effects of school-based physical activity interventions in future studies.

## Electronic supplementary material

Below is the link to the electronic supplementary material.

Supplementary Material 1

## Data Availability

The datasets generated and/or analysed during the current study are available from the corresponding author on reasonable request.
